# An MVA Vector Expressing HIV-1 Envelope under the Control of a Potent Vaccinia Virus Promoter as a Promising Strategy in HIV/AIDS Vaccine Design

**DOI:** 10.3390/vaccines7040208

**Published:** 2019-12-06

**Authors:** Patricia Pérez, María Q. Marín, Adrián Lázaro-Frías, Carlos Óscar S. Sorzano, Mauro Di Pilato, Carmen E. Gómez, Mariano Esteban, Juan García-Arriaza

**Affiliations:** 1 Department of Molecular and Cellular Biology, Centro Nacional de Biotecnología (CNB), Consejo Superior de Investigaciones Científicas (CSIC), 28049 Madrid, Spain; pperez@cnb.csic.es (P.P.); mquiros@cnb.csic.es (M.Q.M.); alazaro@cnb.csic.es (A.L.-F.); cegomez@cnb.csic.es (C.E.G.); 2 Biocomputing Unit, Centro Nacional de Biotecnología (CNB), Consejo Superior de Investigaciones Científicas (CSIC), 28049 Madrid, Spain; coss@cnb.csic.es; 3 Infection and Immunity Group, Istituto di Ricerca in Biomedicina (IRB), Università Della Svizzera Italiana, CH-6500 Bellinzona, Switzerland; mauro.dipilato@irb.usi.ch

**Keywords:** promoter, vaccinia virus, MVA, HIV vaccine, gp120, mice, T and B cell immune responses

## Abstract

Highly attenuated poxviral vectors, such as modified vaccinia virus ankara (MVA), are promising vaccine candidates against several infectious diseases. One of the approaches developed to enhance the immunogenicity of poxvirus vectors is increasing the promoter strength and accelerating during infection production levels of heterologous antigens. Here, we have generated and characterized the biology and immunogenicity of an optimized MVA-based vaccine candidate against HIV/AIDS expressing HIV-1 clade B gp120 protein under the control of a novel synthetic late/early optimized (LEO) promoter (LEO160 promoter; with a spacer length of 160 nucleotides), termed MVA-LEO160-gp120. In infected cells, MVA-LEO160-gp120 significantly increased the expression levels of HIV-1 gp120 mRNA and protein, compared to the clinical vaccine MVA-B vector expressing HIV-1 gp120 under the control of the commonly used synthetic early/late promoter. When mice were immunized with a heterologous DNA-prime/MVA-boost protocol, the immunization group DNA-gp120/MVA-LEO160-gp120 induced an enhancement in the magnitude of gp120-specific CD4^+^ and CD8^+^ T-cell responses, compared to DNA-gp120/MVA-B; with most of the responses being mediated by the CD8^+^ T-cell compartment, with a T effector memory phenotype. DNA-gp120/MVA-LEO160-gp120 also elicited a trend to a higher magnitude of gp120-specific CD4^+^ T follicular helper cells, and modest enhanced levels of antibodies against HIV-1 gp120. These findings revealed that this new optimized vaccinia virus promoter could be considered a promising strategy in HIV/AIDS vaccine design, confirming the importance of early expression of heterologous antigen and its impact on the antigen-specific immunogenicity elicited by poxvirus-based vectors.

## 1. Introduction

The acquired immune deficiency syndrome (AIDS), caused by the human immunodeficiency virus (HIV)-1, has become a pandemic that has spread worldwide, critically affecting human health. According to the Joint United Nations Programme on HIV/AIDS, in 2018, an estimated 1.7 million individuals became newly infected with HIV-1 and 770,000 people died from AIDS-related illnesses worldwide (www.unaids.org). Therefore, the finding of an effective vaccine against HIV/AIDS that could limit the infection is essential to control disease progression within the next years.

An effective vaccine against HIV/AIDS should stimulate both humoral and cellular immune responses to multiple HIV-1 viral antigens, including structural and regulatory proteins, and induce strong, broad, polyfunctional, and durable T- and B-cell responses [1]. Although neutralizing antibodies against gp120 are crucial, due to the difficulty in obtaining immunogens capable of inducing high titers of neutralizing antibodies with broad specificities, a focus on HIV-1-specific T cell immune responses has been one of the main efforts in developing HIV-1 vaccines [2]. For example, in non-human primates, there is a good correlation between vaccine-induced HIV-1-specific cellular immunogenicity and protection after a challenge with a pathogenic simian/human immunodeficiency virus (SHIV) [3,4,5], where CD8^+^ T cells play an important role in immunity to HIV-1 [5]. Moreover, there is substantial evidence that points out that HIV-1-specific CD4^+^ and CD8^+^ T cells mediates protection in vivo [6], and the crucial role played by T cells in HIV-1 suppression comes from studying the immune system in “elite controllers”, a group of people who are able to control HIV-1 replication without any ART treatment [7,8]. Of the numerous clinical trials carried out so far with different HIV/AIDS vaccine candidates, only the RV144 phase III clinical trial showed a modest protection of 31.2% against HIV-1 infection [9]. This clinical trial was based on priming with a recombinant canarypoxvirus ALVAC vector expressing the Env (gp120) protein from subtypes B/E and Gag/Pro from subtype B followed by boosting with HIV-1 gp120 protein from subtypes B/E [9]. Due to the limited efficacy, improved poxvirus recombinants should be considered as components of an effective HIV/AIDS vaccine.

One of the most promising poxvirus vectors is the modified vaccinia virus Ankara (MVA), that has been widely used in numerous preclinical and clinical trials as a vaccine candidate against several prevalent and emerging infectious diseases, including HIV/AIDS, proving to be extremely safe, highly immunogenic, and protective [10,11,12,13,14,15]. Recombinant MVA vectors combine the safety of a killed virus vaccine, due to their impaired replication capacity in mammalian cells, with the immunogenicity of a live virus vaccine. The great efficacy of recombinant MVA vectors in developing antigen-specific immune responses is due to the expression of gene products within cells that are efficiently presented by both MHC class I and class II molecules, leading to the activation of CD4^+^ and CD8^+^ T cells, and to the induction of robust anti-viral responses, as happens with other vaccinia virus (VACV) strains, that make MVA acts as an adjuvant itself [16,17].

Despite the good safety and immunogenicity profiles exhibited by recombinant MVA vectors, novel optimized and more efficient MVA vaccine vectors able to induce an enhanced magnitude, breadth, polyfunctionality, and durability of the immune responses to exogenously expressed antigens are desirable. Thus, several strategies have been developed to enhance the immunogenicity and efficacy of the MVA-based vaccine candidates [18], such as the optimization of the MVA vector itself or the foreign heterologous antigen inserted, the use of optimized prime/boost immunization protocols, or the enhancement of the virus promoter strength. Among them, in this work, we will focus on the optimization of the promoter strength, because it has been shown that the levels of the heterologous antigens expressed (and placed under the control of a VACV promoter) from poxvirus vectors correlates with the magnitude of the antigen-specific immune responses in mice [19]; and timing of antigen expression also influence the type, quantity, quality, and durability of the antigen-specific immune responses [20]. Thus, the optimization of the virus promoter strength is an ideal strategy to increase the expression of the heterologous antigens at very early times post-infection [20,21,22,23]. Moreover, since the efficiency with which an antigen is processed and presented on the surface of infected cells influences its recognition [24], the timing of expression of heterologous antigens from the MVA vector is very important to induce robust antigen-specific T cell immune responses [20]. Considering that immunodominance is defined as the phenomenon whereby only a small fraction of all of the possible epitopes from a particular pathogen elicits an specific immune response [25], it is possible to modulate such immunodominance hierarchy changing the timing and the quantity of antigen production [26]. In fact, it has been described that in VACV, 90% of the most recognized antigens by CD8^+^ T cells were ranked in the top of 50% in terms of mRNA expression [27], and there is a positive correlation between viral gene expression and immunodominance hierarchy after a second immunization due to a mechanism of cross-competition between T cells specific for early and late viral epitopes [28].

Among the different VACV promoters developed that are able to improve the expression and immunogenicity of the foreign antigens, we have previously described a new synthetic late/early optimized (LEO) 160 promoter, designed by using bioinformatic analysis [29,30], that significantly enhance the expression levels of the foreign antigen (green fluorescent protein (GFP) or Leishmania LACK antigen) in vitro, correlating with an in vivo enhancement in the antigen-specific T cell immune responses [29,30]. Since HIV-1 gp120 plays an important role in protective immune responses, the aim of this work is to potentiate the immunogenicity of this antigen through the generation of a novel MVA-based vaccine candidate against HIV/AIDS, containing the optimized stronger VACV LEO160 promoter controlling the expression of HIV-1 gp120 antigen.

## 2. Materials and Methods

### 2.1. Ethics Statement

Female Balb/cOlaHsd mice (6 to 8 weeks old) used for immunogenicity assays were purchased from Envigo Laboratories and stored in the animal facility of the CNB (Madrid, Spain). The immunogenicity animal studies were approved by the Ethical Committee of Animal Experimentation (CEEA) of the CNB (Madrid, Spain) and by the Division of Animal Protection of the Comunidad de Madrid (PROEX 331/14). All animal procedures were conformed to international guidelines and to the Spanish law under the Royal Decree (RD 53/2013).

### 2.2. Cells

DF-1 cells (a spontaneously immortalized chicken embryo fibroblast (CEF) cell line, ATCC catalog no. CRL-12203), primary CEF cells (obtained from specific-pathogen-free 11-day-old eggs; MSD, Salamanca, Spain), and HeLa cells (immortalized human epithelial cervix adenocarcinoma cells, ATCC^®^ CCL-2) were grown in Dulbecco’s modified Eagle’s medium (DMEM) (Gibco-Life Technologies, Carlsbad, CA, USA), supplemented with 10% fetal calf serum (FCS) (Gibco-Life Technologies, Carlsbad, CA, USA) for DF-1 and CEF cells or 10% newborn calf serum (NCS) (Sigma-Aldrich, St. Louis, MO, USA) for HeLa cells. Cell cultures were maintained at 37 °C in a humidified incubator containing 5% CO_2_. Cell lines were infected with viruses, and after 1 h of adsorption, the virus inoculum was removed and DMEM-2% FCS or DMEM-2% NCS was added to the cell cultures.

### 2.3. Viruses

The viruses used in this study included the attenuated MVA wild-type (MVA-WT) strain (kindly provided by G. Sutter) obtained from the chorioallantoic vaccinia virus Ankara strain after 586 serial passages in CEF cells [31], and the recombinant MVA-B expressing HIV-1_IIIB_ Gag-Pol-Nef (GPN) as an intracellular polyprotein and the HIV-1_BX08_ gp120 protein as a cell-released product from HIV-1 clade B isolates [32], which are inserted into the thymidine kinase (TK) locus of the MVA-WT genome under the transcriptional control of a VACV synthetic early/late (sE/L) promoter. MVA-WT was used as the parental virus for the generation of the MVA-LEO160-gp120. All viruses were grown in DF-1 cells to obtain a master seed stock (P2 stock), and titrated in DF-1 cells by plaque immunostaining, using rabbit polyclonal antibody against VACV strain Western Reserve (WR) (diluted 1:1000; Centro Nacional de Biotecnología), followed by an anti-rabbit horseradish peroxidase (HRP)-conjugated secondary antibody (Sigma-Aldrich, St. Louis, MO, USA; diluted 1:1000), as previously described [33]. Determinations of the titers of the different viruses were performed at least two times. Furthermore, viruses grown in primary CEF cells were purified by centrifugation through two 36% (wt/vol) sucrose cushions in 10 mM Tris-HCl pH 9. All viral stocks were free of contamination with mycoplasma (checked by specific polymerase chain reaction (PCR) for mycoplasma), bacteria (checked by growth in LB plates without ampicillin), or fungi (checked by growth in Columbia blood agar plates; Oxoid).

### 2.4. Construction of Plasmid Transfer Vector pLZAW1-LEO160-gp120

This plasmid was used for the insertion of the HIV-1 gp120 sequence (clade B, isolate BX08, GenBank accession number: GQ855765.1), under the control of the novel synthetic VACV LEO160 promoter into the TK locus of MVA. It was generated by inserting the HIV-1_Bx08_ gp120 sequence into the pLZAW1-LEO160 plasmid [29], that contains the novel synthetic VACV LEO160 promoter by using GeneArt Subcloning and Plasmid Services (Thermo Fisher Scientific, Waltham, MA, USA). Thus, pLZAW1-LEO160-gp120 contains the HIV-1_BX08_ gp120 sequence under the control of the novel synthetic VACV LEO160 promoter introduced in a multiple cloning site between the MVA TK left (TK-L) and TK right (TK-R) flanking regions, and the selectable marker genes for ampicillin and β-galactosidase (β-gal). The β-gal gene (LacZ) is inserted among two repetitions of the TK-L flanking region, allowing their deletion from the final recombinant virus by homologous recombination after consecutive plaque purification steps. The resulting plasmid pLZAW1-LEO160-gp120 (9217 bp) was confirmed by DNA sequence analysis.

### 2.5. Generation of MVA-LEO160-gp120 Recombinant Virus

The MVA-LEO160-gp120 recombinant virus was generated using MVA-WT as parental virus, and pLZAW1-LEO160-gp120 as plasmid transfer vector; employing an infection/transfection protocol previously described [34,35,36,37,38,39]. After the infection/transfection in DF-1 cells, we initially selected blue plaques stained with 5-bromo-4-chloro-3-indolyl-beta-d-galactopyranoside (X-Gal, Sigma-Aldrich, St. Louis, MO, USA). In the first three passages viruses from selected blue plaques expressing β-Gal were picked, and in the last three passages (six passages in total) viruses from selected plaques do not express any marker due to the loss of β-Gal marker. The isolated plaques were expanded in DF-1 cells until cytopathic effect was observed, and then crude viral extracts obtained were used for the next plaque purification round. After these recombination events in cell culture, the final plaques were selected and expanded in DF-1 cells to obtain a master seed stock (P2 stock).

### 2.6. PCR Analysis

To verify that the HIV-1 gp120 sequence under the control of the novel synthetic VACV LEO160 promoter was correctly inserted in MVA-LEO160-gp120, viral DNA was extracted from DF-1 cells mock infected or infected at 5 plaque forming units (PFU)/cell with the different viruses, as previously described [34], and the correct insertion was confirmed by PCR analysis. Primers TK-L (TGATTAGTTTGATGCGATTC) and TK-R (TGTCCTTGATACGGCAG) spanning the MVA TK locus, were used for PCR analysis, to verify the correct insertion of the LEO160-gp120 sequence in MVA-LEO160-gp120. The insertion was also confirmed by DNA sequence analysis (Secugen, Madrid, Spain). The amplification protocols were performed using PuReTaq™ Ready-To-Go™ PCR beads (GE Healthcare, Chicago, IL, USA), in accordance with the manufacturer’s protocol. PCR products were run in 1% agarose gel and visualized by SYBR Safe staining (Invitrogen, Carlsbad, CA, USA).

### 2.7. Analysis of Virus Growth

To study the virus growth profile of MVA-LEO160-gp120, monolayers of DF-1 cells grown in 12-well plates were infected in duplicate at 0.01 PFU/cell with MVA-WT and MVA-LEO160-gp120. Following virus adsorption for 60 min at 37 °C, the inoculum was removed. The infected cells were washed with DMEM and incubated with fresh DMEM containing 2% FCS at 37 °C in a 5% CO_2_ atmosphere. At different times (0, 24, 48, and 72 h post-infection (hpi)), cells were collected by scraping, freeze-thawed 3 times, and briefly sonicated. Virus titers in cell lysates were determined by immunostaining plaque assay as previously described [33].

### 2.8. Expression of HIV-1_BX08_ gp120 by Western Blot

To check the correct expression of the HIV-1_BX08_ gp120 protein, monolayers of DF-1 cells were mock infected or infected at 5 PFU/cell with the different viruses. At 24 hpi, cell extracts were lysed in Laemmli buffer and fractionated in 8% SDS-PAGE, and then analyzed by Western blotting with rabbit polyclonal anti-gp120 antibody against clade B IIIB strain (CNB; diluted 1:3000) to analyze the expression of the gp120 protein. As loading controls, we used rabbit anti-β-actin (Cell Signaling, Danvers, MA, USA; diluted 1:1000), and rabbit anti-VACV E3 (CNB; diluted 1:1000) antibodies. An HRP-conjugated anti-rabbit antibody (Sigma-Aldrich, St. Louis, MO, USA; diluted 1:5000) was used as the secondary antibody. The immunocomplexes were detected with an HRP-luminol enhanced-chemiluminescence system (ECL Plus) (GE Healthcare, Chicago, IL, USA).

### 2.9. Genetic Stability of Recombinant MVA-LEO160-gp120 by Expression Analysis

The genetic stability of recombinant MVA-LEO160-gp120 was analyzed as previously described [34,40]. MVA-LEO160-gp120 (P2 stock) was continuously grown at low multiplicity of infection (MOI) in DF-1 cells during 9 passages and then 28 individual plaques were picked from virus derived from passage 9. Next, viruses from the 9 passages and the 28 individual plaques from passage 9 were used to infect DF-1 cells and the expression of HIV-1_BX08_ gp120 protein was checked by Western Blot as described above.

### 2.10. RNA Analysis of HIV-1 gp120 by Reverse Transcription Real-Time Quantitative PCR (RT-qPCR)

Total RNA was isolated using the RNeasy Kit (Qiagen, Hilden, Germany), from non-permissive HeLa or permissive DF-1 cells mock infected or infected at 5 PFU/cell with the different viruses and harvested at different times post-infection. Reverse transcription of maximum 1000 ng of RNA was performed with the QuantiTect reverse transcription kit (Qiagen, Hilden, Germany), according to the manufacturer’s recommendations. Quantitative PCR was performed with a 7500 Real-Time PCR system (Applied Biosystems, Foster City, CA, USA) using Power SYBR green PCR Master Mix (Applied Biosystems, Foster City, CA, USA), as previously described [17]. The mRNA expression levels of HIV-1 gp120 was analyzed by real-time PCR with specific oligonucleotides (sequences are available upon request). Specific gene expression was expressed relative to the expression of the cellular hypoxanthine phosphoribosyltransferase (HPRT) gene in arbitrary units (AU) using the 2−ΔΔCt method [41]. All samples were tested in triplicate, and 2 independent experiments were performed.

### 2.11. Expression Kinetics of HIV-1_BX08_ gp120 by Western Blot

To compare the expression kinetics of the HIV-1_BX08_ gp120 protein between MVA-B and MVA-LEO160-gp120 viral vectors, monolayers of DF-1 cells were mock infected or infected at 5 PFU/cell with the different viruses. At 2, 4, 6, and 24 hpi, cell extracts were obtained and quantified using Pierce BCA Protein Assay Kit (Thermo Fisher Scientific, Waltham, MA USA), following manufacturer´s recommendations. Next, 30 μg of each cell extract was lysed in Laemmli buffer, fractionated in 8% SDS-PAGE, and then analyzed by Western blotting with rabbit polyclonal anti-gp120 antibody against clade B IIIB strain (CNB; diluted 1:3000). As viral loading control we used a rabbit anti-VACV E3 (CNB; diluted 1:1000) antibody. An HRP-conjugated anti-rabbit antibody (Sigma-Aldrich, St. Louis, MO, USA; diluted 1:5000) was used as the secondary antibody. The immunocomplexes were detected with an HRP-luminol enhanced-chemiluminescence system (ECL Plus) (GE Healthcare, Chicago, IL, USA), according to the manufacturer´s instructions, and detected in a ChemiDoc™ Imaging System (Bio-Rad). Band intensities were quantified using Image Lab software (Bio-Rad, Hercules, CA, USA) and gp120:E3 intensity ratios were represented and analyzed using GraphPad Prism Software (San Diego, CA, USA).

### 2.12. HIV-1_BX08_ gp120 Protein Quantification by Enzyme Linked Immunosorbent Assay (ELISA)

To quantify the concentration of HIV-1_BX08_ gp120 protein secreted to supernatants from infected cells, 100 mm diameter culture dishes (Falcon) of HeLa cells were infected with the corresponding MVA recombinant viruses and incubated with 12 ml of DMEM-Hi glucose medium (Sigma-Aldrich, St. Louis, MO, USA) for 2, 4, 6, and 24 h at 37 °C. Then, supernatants were centrifuged at 1500 rpm for 5 min to clarify them and concentrated using Amicon^®^ Ultra-15 Centrifugal Filters (Millipore, Burlington, MA, USA). Next, 96-well plates (NUNC MaxiSorp™, Thermo Fisher Scientific, Waltham, MA, USA) were coated with concentrated supernatants and, at the same time, with serial dilutions (in PBS) of the purified HIV-1_BX08_ gp120 protein (CNB) at known concentrations in order to have a standard curve. Plates were incubated at 4 °C overnight and, the next day, blocked for 1 h with 5% skimmed milk prepared in PBS-T, followed by three washes with PBS-T. Next, the protein coated plates were incubated for 2 h at room temperature (RT) with primary antibody anti-HIV-1 gp120 2G12 (NIAID-NIH) diluted 1:100 in PBS-1% skimmed milk-0.01%Tween20. Then, the plates were washed three times again with PBS-T and incubated for 1 h at RT with secondary antibody goat anti-human-HRP (Sigma-Aldrich, St. Louis, MO, USA) diluted 1/1000 in PBS-1% skimmed milk-0.01%Tween20. Finally, after another washing step, the plates were developed by adding 100 µL of 3,3′,5,5′ Tetramethylbenzidine (TMB) substrate (Sigma-Aldrich, St. Louis, MO, USA) and the reaction was stopped by adding 50 µL of 1 M H_2_SO_4_. The absorbance was read at 450 nm using an EZ Read 400 microplate reader (Biochrom Ltd., Cambourne, Cambridge, UK).

### 2.13. DNA Vectors

DNA plasmids expressing HIV-1_BX08_ gp120 (pCMV-gp120_BX08_) and the empty plasmid (pCMV-ϕ) have been previously described [32] and were purified with the EndoFree Plasmid Mega kit (Qiagen, Hilden, Germany) in accordance with the manufacturer’s protocol and diluted for injection in endotoxin-free phosphate-buffered saline (PBS) (Gibco-Life Technologies, Carlsbad, CA, USA). pCMV-ϕ (termed DNA-ϕ) or pCMV-gp120_BX08_ (termed DNA-gp120) have been used in the immunization protocol as a prime.

### 2.14. Peptides

HIV-1 peptide pools, with each purified peptide at 1 mg/mL per vial, were provided by BEI Resources, NIH. The peptides covered the Env protein present in the consensus sequence of HIV-1 clade B (gp120 from isolate BX08) as consecutive 15-mers overlapping by 11 amino acids. The HIV-1_BX08_ gp120 protein was spanned by the Env-1 and Env-2 peptide pools.

### 2.15. Mouse Immunization Schedule

Female BALB/c mice (6 to 8 weeks old) were purchased from Envigo Laboratories and stored in a pathogen-free barrier area of the CNB in accordance to the recommendations of the Federation of European Laboratory Animal Science Associations. A DNA prime/MVA boost immunization protocol was performed as previously described [32,37,38,39,42] to assay the immunogenicity of MVA-LEO160-gp120. Groups of animals (*n* = 5) received 100 μg of DNA-gp120 (100 μg of pCMV-gp120_BX08_) or 100 μg of DNA-ϕ (100 μg of pCMV-ϕ) in 50 μL of PBS by the intramuscular (i.m.) route and 2 weeks later received an intraperitoneal (i.p.) inoculation of 1 × 10^7^ PFU of the corresponding MVA virus (MVA-WT, MVA-B, or MVA-LEO160-gp120) in 200 μL of PBS. Mice primed with sham DNA (DNA-ϕ) and boosted with nonrecombinant MVA-WT were used as a control group. At 10 days after the last immunization, mice were sacrificed with carbon dioxide (CO_2_) and their spleens and blood samples were processed to measure the adaptive T cell and humoral immune responses to HIV-1 gp120, respectively, by using intracellular cytokine staining (ICS) assay or enzyme-linked immunosorbent assay (ELISA). Two independent experiments were performed.

### 2.16. ICS Assay

The magnitude, breadth, polyfunctionality, and phenotype of the HIV-1-specific T cell adaptive immune responses were analyzed by ICS as previously described [34,37,38,39,43], with some modifications. After spleen processing, fresh 4 × 10^6^ splenocytes (depleted of red blood cells) were seeded onto M96 plates and stimulated for 6 h in complete RPMI 1640 medium supplemented with 10% FCS containing 1 μL/mL Golgiplug (BD Biosciences, Franklin Lakes, NJ, USA) to inhibit cytokine secretion; monensin 1X (eBioscience, Thermo Fisher Scientific, Waltham, MA, USA), anti-CD107a–FITC (BD Biosciences, Franklin Lakes, NJ, USA); and HIV-1 Env peptide pools (5 μg/mL). Then, cells were washed, stained for the surface markers, fixed, permeabilized (Cytofix/Cytoperm kit; BD Biosciences, Franklin Lakes, NJ, USA), and stained intracellularly with the appropriate fluorochromes. Dead cells were excluded with the violet LIVE/DEAD stain kit (Invitrogen, Carlsbad, CA, USA). The fluorochrome-conjugated antibodies used for functional analyses were CD3-phycoerythrin (PE)-CF594, CD4-allophycocyanin (APC)-Cy7, CD8-V500, IFN-γ–PE-Cy7, TNF-α–PE, and IL-2–APC. In addition, the antibodies used for phenotypic analyses were CD62L-Alexa 700 and CD127-peridinin chlorophyll protein (PerCP)-Cy5.5. All antibodies were from BD Biosciences, Franklin Lakes, NJ, USA.

The magnitude of the HIV-1-specific T follicular helper (Tfh) cell adaptive immune responses was analyzed by ICS as previously described [44,45], with some modifications. After spleen processing, fresh, 4 × 10^6^ splenocytes (depleted of red blood cells) were seeded onto M96 plates using RPMI-10% FCS and stimulated with 5 µg/mL of Env peptide pools and 0.5 µg/mL of HIV-1 gp120 envelope protein from isolate BX08 (CNB) along with anti-CD154 (CD40L)-PE antibody at 37 °C. Two hours later, 1 µL/mL protein transport inhibitor GolgiPlug (BFA, BD Biosciences, Franklin Lakes, NJ, USA), and monensin (1X; eBioscience, Thermo Fisher Scientific, Waltham, MA, USA), were added and cells were keep incubated for 4 additional hours at 37 °C. Next, live cells were stained using fixable viability stain (FVS) 520 (BD Biosciences, Franklin Lakes, NJ, USA) for 20 min at 4 °C. Then, after being washed twice with IB buffer (PBS 1X-FCS 2%-EDTA 2 mM), cells were stained for the surface markers using 50 μL of the corresponding antibodies CD4-Alexa 700, CD44-PECy5, CXCR5-PE-CF594, PD1(CD279)-APC-eFluor780 and CD8-V500 diluted following manufacturer’s instructions for 20 min at 4 °C. After being washed again two times with IB buffer, splenocytes were fixed and permeabilized with BD Cytofix/Cytoperm™ solution Kit (BD Biosciences, Franklin Lakes, N.J., USA) for 20 min at 4 °C and rested overnight in IB buffer. The day after, cells were washed with Permwash 1X (BD Biosciences, Franklin Lakes, NJ, USA) and the Fc receptors were blocked with 25 μL of an anti CD16/CD32 (FcBlock) antibody (diluted 1:100 in Permwash 1×) for 5 min at 4 °C. Finally, the cells were stained intracellularly for cytokines using 25 μL of intracellular antibodies IL-4-FITC, IFNγ-PECy7, and IL-21-APC (diluted following manufacturer’s instructions) for 20 min at 4 °C and washed then twice in Permwash 1X after resuspended them in 200 μL of IB buffer.

Cells were acquired with a Gallios flow cytometer (Beckman Coulter, Brea, CA, USA). Data analysis was carried out using FlowJo software (version 8.5.3, Tree Star, Ashland, OR, USA). After gating, boolean combinations of single functional gates were created with the FlowJo software to determine the frequency of each response based on all possible combinations of cytokine expression or all possible combinations of differentiation marker expression. Background responses detected in negative-control samples were subtracted from those detected in stimulated samples for every specific functional combination.

### 2.17. Antibody Measurements by ELISA

The total IgG anti-HIV-1_BX08_ gp120 envelope protein antibodies in pooled sera from immunized mice were measured by ELISA, as previously described [32,42].

### 2.18. Statistical Procedures

Student’s t test was used for analysis of gp120 transgene expression and for protein and antibody measurements to establish the differences between two groups. Statistical analysis of the ICS assay results was realized, as previously described [42,43], by an approach that corrects measurements for the medium response (RPMI), calculating confidence intervals and *P* values. Only antigen response values significantly larger than the corresponding RPMI are presented. Background values were subtracted from all of the values used to allow analysis of proportionate representation of responses. The statistical significances are indicated as follows: *, *p* < 0.05; **, *p* < 0.005; ***, *p* < 0.001.

## 3. Results

### 3.1. Generation and In Vitro Characterization of MVA-LEO160-gp120

**Figure 1:**
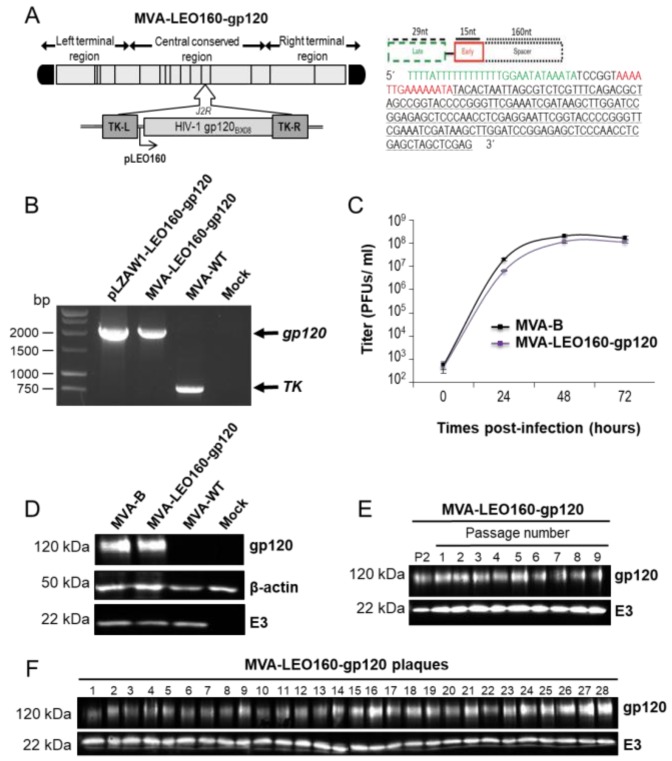
Generation and in vitro characterization of modified vaccinia virus Ankara (MVA)- late/early optimized (LEO)160-gp120. (**A**) (left panel) Scheme of the MVA-LEO160-gp120 genome map. The HIV-1 gp120 gene (from the clade B, isolate BX08) placed under the control of the LEO160 promoter and inserted within the MVA thymidine kinase (TK) viral locus (*J2R* gene) is indicated. TK-L = TK left flanking region, TK-R = TK right flanking region. (**A**) (right panel) Scheme and sequence of the synthetic LEO160 promoter. Late promoter element (29 nucleotides; green); early promoter motif (15 nucleotides; red), and a 160-nucleotide spacer (consisting in several multicloning sites; black) are shown. Adapted from [29]. (**B**) PCR analysis of MVA TK locus. Viral DNA was extracted from DF-1 cells mock infected or infected at 5 PFU/cell with MVA-WT, or MVA-LEO160-gp120. DNA from pLZAW1-LEO160-gp120 plasmid transfer vector was used as positive control for LEO160-gp120 insert. Primers spanning the TK-L and TK-R flanking regions were used for PCR analysis. DNA products corresponding to the MVA TK gene and the LEO160-gp120 insertion are indicated on the right. Molecular size markers (1-kb ladder) with the corresponding sizes (base pairs) are indicated on the left. (**C**) Viral growth kinetics. Monolayers of permissive DF-1 cells were infected at 0.01 PFU/cell with MVA-B or MVA-LEO160-gp120. At different times post-infection (0, 24, 48, and 72 h post-infection (hpi)) cells were collected and virus titers in cell lysates were quantified by plaque immunostaining assay with anti-vaccinia virus (VACV) antibodies. The mean ± standard deviations of two independent experiments are shown. (**D**) Expression of HIV-1 gp120 protein. Western blot analysis of the HIV-1 gp120 protein detected in cells extracts of DF-1 cells mock infected or infected with MVA-B, MVA-LEO160-gp120, or MVA-WT. Antibodies against VACV E3 and β-actin were used as viral and cellular loading controls, respectively. The proteins detected are indicated on the right and their protein molecular weights (in kDa) are indicated on the left. (**E**,**F**) Stability of MVA-LEO160-gp120. MVA-LEO160-gp120 (P2 stock) was continuously grown at low MOI in DF-1 cells to passage 9 and at passage 9, 28 individual plaques were picked. Virus stocks from each passage (**E**) and from the 28 individual plaques at passage 9 (**F**) were used to infect cells and the expression of HIV-1 gp120 protein was determined by Western blotting. Rabbit anti-VACV E3 protein antibody was used as a VACV loading control. The proteins detected are indicated on the right and their protein molecular weights (in kDa) are indicated on the left.

To study if the novel LEO160 promoter was also able to increase the expression levels and the immune responses of a soluble antigen, such as the HIV-1 gp120 protein, in comparison with the HIV/AIDS vaccine candidate MVA-B that expresses HIV-1 gp120 under the VACV sE/L promoter [32], a novel MVA vector expressing the HIV-1 envelope gp120 protein (clade B, isolate BX08) under the control of the synthetic VACV LEO160 promoter was generated (termed MVA-LEO160-gp120), as described in Materials and Methods (Figure 1A).

The correct presence of the VACV LEO160 promoter and the HIV-1 gp120 gene in the MVA-LEO160-gp120 recombinant virus was analyzed by PCR using oligonucleotides annealing in the VACV TK-flanking regions (Figure 1B) and was also confirmed by DNA sequencing. The virus growth kinetics in cultured permissive chicken DF-1 cells of the novel MVA-LEO160-gp120 recombinant virus and MVA-B (used as control) were similar (Figure 1C), proving that the insertion of the VACV LEO160 promoter and the HIV-1 gp120 gene does not affect MVA vector replication under permissive conditions. The correct expression of the heterologous HIV-1 gp120 protein was studied by Western blot in cell extracts from DF-1 cells, mock infected or infected with MVA-LEO160-gp120, MVA-B, or MVA-WT using a specific rabbit polyclonal anti-gp120 antibody. The results demonstrated that MVA-LEO160-gp120 correctly expressed the HIV-1 gp120 protein (Figure 1D). Moreover, to ensure that the encoded HIV-1 gp120 protein is stably expressed from the MVA genome and its expression can be maintained though long-time passages, MVA-LEO160-gp120 was grown in DF-1 cells infected at low MOI (0.01 PFU/cell) for nine consecutive passages (Figure 1E) and at passage 9, 28 individual virus plaques were isolated (Figure 1F). The expression of the HIV-1 gp120 protein was determined in cell extracts by Western blot, revealing that MVA-LEO160-gp120 efficiently expresses the HIV-1 gp120 protein at all passages (Figure 1E) and that 100% of the plaques at passage 9 correctly expressed the HIV-1 gp120 protein (Figure 1F), demonstrating the high genetic stability of MVA-LEO160-gp120.

### 3.2. MVA-LEO160-gp120 Increases the Expression and Cell Release of HIV-1 Envelope gp120 Antigen

**Figure 2:**
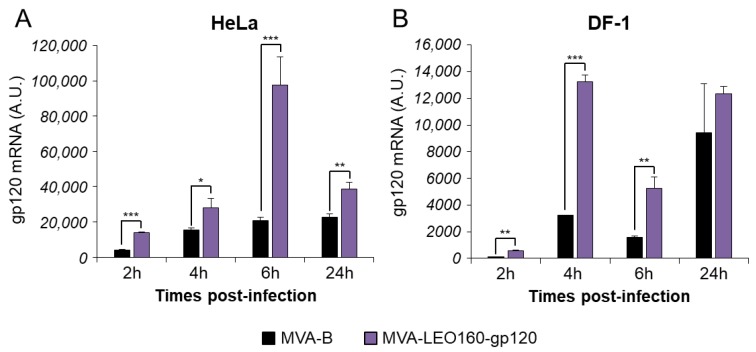
MVA-LEO160-gp120 enhances the mRNA levels of HIV-1 gp120, compared to MVA-B. HeLa (**A**) or DF-1 cells (**B**) were mock infected or infected with MVA-B, or MVA-LEO160-gp120 at 5 PFU/cell. At 2, 4, 6, and 24 hpi, RNA was extracted, and HIV-1 gp120 expression was analyzed by RT-qPCR. Results are expressed as the ratio of HIV-1 gp120 to endogenous HPRT mRNA levels. A.U. = arbitrary units. *P* values indicate significant response differences between MVA-B and MVA-LEO160-gp120 at the same hour (*, *p* < 0.05; **, *p* < 0.005; ***, *p* < 0.001). Data are means ± standard deviations of triplicate samples from one experiment and are representative of two independent experiments.

To determine whether MVA-LEO160-gp120 could enhance the expression levels of HIV-1 gp120, in comparison to MVA-B, HeLa or DF-1 cells were infected with MVA-B and MVA-LEO160-gp120 at a MOI of 5 PFU/cell for 2, 4, 6, and 24 h. Then, total RNA was isolated and mRNA levels of HIV-1 gp120 were determined by RT-qPCR. The results showed that MVA-LEO160-gp120 significantly increased HIV-1 gp120 transcription compared to MVA-B in either HeLa (Figure 2A) or DF-1 cells (Figure 2B). Noticeable are the differences observed in mRNA levels at early times post infection, highlighting the robust increase in gene expression achieved by the LEO160 promoter.

**Figure 3:**
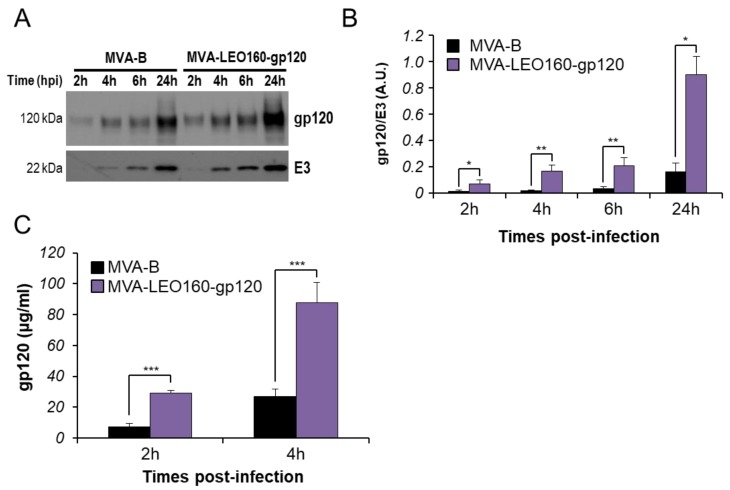
MVA-LEO160-gp120 enhances the protein levels of HIV-1 gp120, compared to MVA-B. (**A**) HIV-1 gp120 protein expression in HeLa cells infected at 5 PFU/cell with MVA-B or MVA-LEO160-gp120 at 2, 4, 6, and 24 hpi. VACV E3 protein expression was used as a VACV loading control. (**B**) Bars showed the ratio of HIV-1 gp120 protein to VACV E3, after quantification of the corresponding band intensities represented in panel A, using Image Lab software. A.U. = arbitrary units. Values showed the mean ± SEM of two independent experiments. *P* values indicate significant response differences between MVA-B and MVA-LEO160-gp120 at the same hour (*, *p* < 0.05; **, *p* < 0.005). (**C**) Levels of HIV-1 gp120 protein secreted to the supernatant. HeLa cells were infected at 5 PFU/cell with MVA-B or MVA-LEO160-gp120 and at 2 and 4 hpi supernatants were concentrated and the amount of HIV-1 gp120 protein was determined by ELISA. Data are means ± standard deviations of duplicate samples from one experiment and are representative of two independent experiments. *P* values indicate significant response differences between MVA-B and MVA-LEO160-gp120 at the same hour (***, *p* < 0.001).

Next, in order to compare the HIV-1 gp120 protein expression between MVA-B and MVA-LEO160-gp120, total protein was extracted at different time points (2, 4, 6, and 24 h) from HeLa cells infected at 5 PFU/cell with MVA-B or MVA-LEO160-gp120. Next, 30 μg of protein were loaded on SDS-PAGE and the HIV-1 gp120 protein levels were detected by Western blot (Figure 3A). The results showed that MVA-LEO160-gp120 increased the expression levels of HIV-1 gp120 protein, compared to MVA-B (Figure 3A). Furthermore, the band intensity was quantified using Image Lab software and the expression of HIV-1 gp120 protein was normalized to VACV E3 protein (VACV constitutive early protein) to show that the difference in heterologous antigen expression was the result of distinct promoter strengths, and not to different virus infective capacities. The results showed that MVA-LEO160-gp120 induced a significantly increased in gp120 production compared to MVA-B at all time points analyzed (Figure 3B), correlating with the previous results of mRNA levels (Figure 2).

To further analyze whether there were differences in cell released of soluble HIV-1 gp120 protein to the extracellular medium, supernatants derived from HeLa cells infected with MVA-B or MVA-LEO160-gp120 were collected at 2, 4, 6, and 24 hpi and concentrated using Amicon^®^ Ultra-15 Centrifugal Filters (Millipore, Burlington, MA, USA). The total amount of HIV-1 gp120 protein present in the supernatants was quantified by ELISA using a standard curve of purified HIV-1 gp120_BX08_ protein. The results showed that MVA-LEO160-gp120 released more soluble HIV-1 gp120 to the extracellular medium than MVA-B at early times post-infection (2 and 4 h) (Figure 3C), but at later times post-infection (6 and 24 h), there were no significant differences between MVA-LEO160-gp120 and MVA-B in the total amount of HIV-1 gp120 protein released to the supernatant, probably mediated by cytopathic and apoptosis induction.

These in vitro results with virus-infected cells confirmed that the VACV LEO160 promoter positively enhances the expression of the antigen HIV-1 gp120.

### 3.3. MVA-LEO160-gp120 Increases the Magnitude of Env-Specific T Cell Immune Responses in Mice

To determine whether the increased HIV-1 gp120 early expression observed in vitro in cells infected with MVA-LEO160-gp120 could drive an enhancement in the Env-specific T cell responses in vivo, the HIV-1 Env-specific CD4^+^ and CD8^+^ T cell immune responses induced in mice immunized with MVA-B and MVA-LEO160-gp120 were analyzed. A DNA prime/MVA boost immunization protocol was used, as this protocol amplifies the levels of T and B cell responses compared to the homologous MVA prime/MVA boost immunization [4,32]. Thus, mice received 100 μg of DNA-gp120 prime by i.m. route and 14 days later were boosted with 1 × 10^7^ PFU of MVA viruses (MVA-B or MVA-LEO160-gp120) by i.p. route. Animals primed with sham DNA (DNA-ϕ) and boosted with non-recombinant MVA-WT were used as a control group. Adaptive Env-specific CD4^+^ and CD8^+^ T cell immune responses elicited by the different immunization groups (DNA-gp120/MVA-B, DNA-gp120MVA-LEO160-gp120, and DNA-ϕ/MVA-WT) were measured 10 days after the boost by ICS assay, after the stimulation of splenocytes with a pool of Env peptides that spanned the HIV-1 gp120 from an HIV-1 clade B consensus sequence.

**Figure 4:**
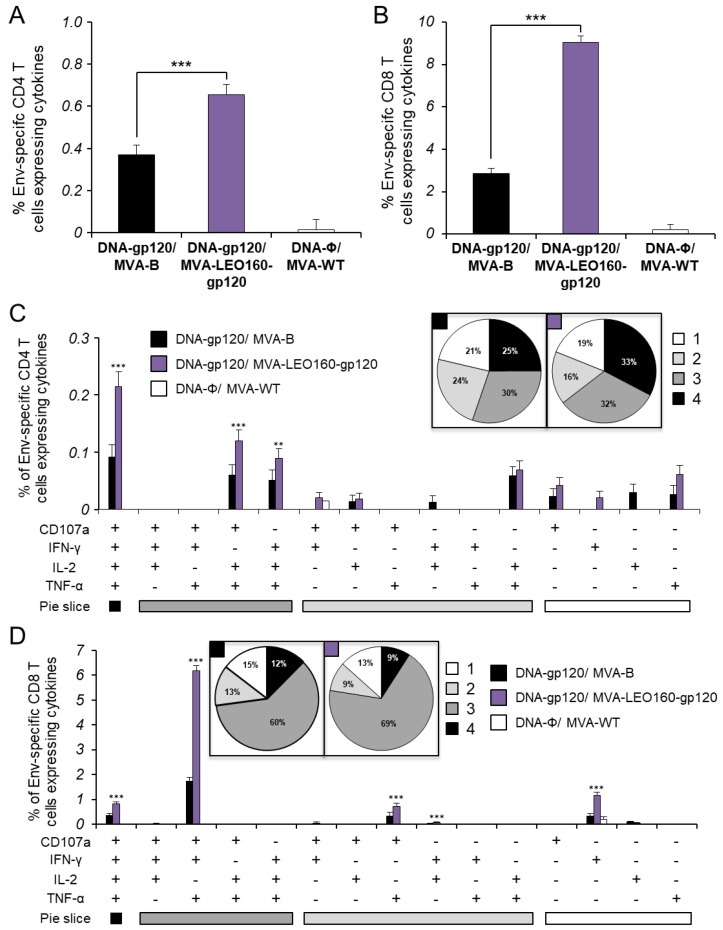
Immunization with DNA-gp120/MVA-LEO160-gp120 enhances the magnitude of HIV-1 Env-specific CD4^+^ and CD8^+^ T cell adaptive immune responses. Splenocytes were collected from mice (n = 5 per group) immunized with DNA-ϕ/MVA-WT, DNA-gp120/MVA-B, or DNA-gp120/MVA-LEO160-gp120, 10 days after the last immunization. Next, HIV-1 Env-specific CD4^+^ and CD8^+^ T cell adaptive immune responses triggered by the different immunization groups were measured by ICS assay following the stimulation of splenocytes with an Env peptide pool (comprising Env-1 + Env-2 peptide pools). Values from unstimulated controls were subtracted in all cases. *P* values indicate significant response differences between the DNA-gp120/MVA-B and DNA-gp120/MVA-LEO160-gp120 immunization groups (**, *p* < 0.005; ***, *p* < 0.001). (**A**,**B**) Overall percentages of Env-specific CD4^+^ (**A**) and CD8^+^ (**B**) T cells. The values represent the sum of the percentages of T cells expressing CD107a and/or IFN-γ and/or TNF-α and/or IL-2 against the Env peptide pool. (**C**,**D**) Polyfunctional profiles of Env-specific CD4^+^ (**C**) and CD8^+^ (**D**) T cells. All of the possible combinations of responses are shown on the x axis, while the percentages of T cells expressing CD107a and/or IFN-γ and/or TNF-α and/or IL-2 against the Env peptide pool are shown on the y axis. Responses are grouped and color coded on the basis of the number of functions (4, 3, 2, or 1). The pie charts summarize the data. Each slice corresponds to the proportion of the total Env-specific CD4^+^ and CD8^+^ T cells exhibiting 1, 2, 3, or 4 functions (CD107a and/or IFN-γ and/or TNF-α and/or IL-2).

The magnitude of the total HIV-1 Env-specific CD4^+^ (Figure 4A) and CD8^+^ (Figure 4B) T cell adaptive immune responses (determined as the sum of the individual responses expressing IFN-γ, TNF-α, and/or IL-2 cytokines, as well as the expression of CD107a on the surface of activated T cells as an indirect marker of cytotoxicity) was significantly greater in the DNA-gp120/MVA-LEO160-gp120 immunization group than in DNA-gp120/MVA-B, with both vaccinated groups triggering an overall Env-specific immune response mediated mainly by CD8^+^ T cells (Figure 4A,B).

Furthermore, the quality of the Env-specific T cell adaptive immune responses was characterized in part by the pattern of cytokine production and its cytotoxic potential. Thus, on the basis of the production of CD107a, IFN-γ, TNF-α, and IL-2 from HIV-1 Env-specific CD4^+^ and CD8^+^ T cells, 15 different HIV-1 Env-specific CD4^+^ and CD8^+^ T cell populations could be identified (Figure 4C,D). As shown in Figure 4C (pie charts), Env-specific CD4^+^ T cell responses were similarly polyfunctional in both vaccinated groups, with around 80% of the CD4^+^ T cells exhibiting two or more functions. CD4^+^ T cells expressing CD107a+IFN-γ+TNF-α+IL-2, CD107a+TNF-α+IL-2 or IFN-γ+TNF-α+IL-2 were the most induced populations elicited by both vaccinated groups, but DNA-gp120/MVA-LEO160-gp120 induced a significantly greater percentage of these major populations than DNA-gp120/MVA-B (Figure 4C, bars). On the other hand, as shown in Figure 4D (pie charts), DNA-gp120/MVA-B and DNA-gp120/MVA-LEO160-gp120 have a similar polyfunctional profile of Env-specific CD8^+^ T cell responses, with 85% and 87% of the CD8^+^ T cells exhibiting two or more functions, respectively. CD8^+^ T cells expressing CD107a+IFN-γ+TNF-α was the most abundant population elicited by both vaccinated groups, but once again DNA-gp120/MVA-LEO160-gp120 induced a significantly greater increase in the percentage of this population, and others, than DNA-gp120/MVA-B (Figure 4D, bars).

### 3.4. MVA-LEO160-gp120 Enhances the Magnitude of Env-Specific T Cells with an Effector Memory Phenotype

**Figure 5:**
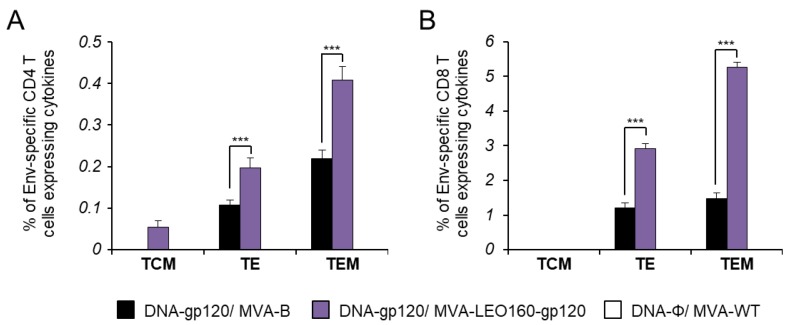
Phenotypic profile of Env-specific CD4^+^ and CD8^+^ T cells. Percentages of T central memory (TCM), T effector memory (TEM), and T effector (TE) HIV-1 Env-specific CD4^+^ (**A**) and CD8^+^ (**B**) T cells expressing CD107a and/or IFN-γ and/or TNF-α and/or IL-2 against Env peptide pool 10 days after the last immunization (adaptive phase). Values from unstimulated controls were subtracted in all cases. *P* values indicate significant response differences between the DNA-gp120/MVA-B and DNA-gp120/MVA-LEO160-gp120 immunization groups (***, *p* < 0.001).

It has been described that HIV-1-specific T cells of a mature effector memory phenotype are more frequently detectable in HIV-1 controllers than in HIV-1 progressors [46,47,48]. Thus, next we determined the memory phenotype of HIV-1 Env-specific CD4^+^ and CD8^+^ T cells by measuring the expression of the CD127 and CD62L surface markers, which allow the definition of the different memory subpopulations: T central memory (TCM, CD127^+^/CD62L^+^), T effector memory (TEM, CD127^+^/CD62L^−^), and T effector (TE, CD127^−^/CD62L^−^) cells [49], and determined as the sum of the individual responses expressing CD107a, IFN-γ, TNF-α, and/or IL-2 obtained for the Env peptide pool (Figure 5). The results showed that in both vaccinated groups, Env-specific CD4^+^ and CD8^+^ T cells were mainly of the TEM phenotype, followed by the TE phenotype. However, immunization with DNA-gp120/MVA-LEO160-gp120 induced a significantly greater increase in the percentage of Env-specific CD4^+^ and CD8^+^ TEM and TE cells than immunization with DNA-gp120/MVA-B (Figure 5). 

### 3.5. MVA-LEO160-gp120 Increases the Magnitude of Env-Specific CD4^+^ T Follicular Helper (Tfh) Cell Responses

**Figure 6:**
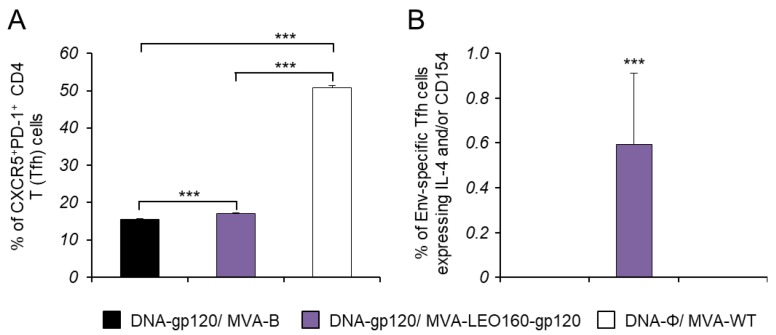
Env-specific Tfh cell immune responses. Mice (n = 5) were immunized with DNA-gp120/MVA-B, DNA-gp120/MVA-LEO160-gp120, or DNA-ϕ/MVA-WT. At 10 days after the last immunization, levels of CD4^+^ Tfh cells and Env-specific CD4^+^ Tfh cell immune response was studied in splenocytes by ICS assay. *P* values indicate significant response differences between immunization groups (***, *p* < 0.001). (**A**) Magnitude of the CD4^+^ T cells with Tfh phenotype (CXCR5^+^, PD1^+^) measured in non-stimulated (RPMI) splenocytes. (**B**) Magnitude of Env-specific CD4^+^ Tfh cells. The total value in each group represents the sum of the percentages of CD4^+^ Tfh cells expressing IL-4 and/or CD154 against gp120_BX08_ protein plus Env peptide pool. Data are background (RPMI)-subtracted.

The development of HIV-1 broadly neutralizing antibodies (bNAbs) has been previously correlated with the frequency and quality of CD4^+^ T follicular helper (Tfh) cells [50,51]. This subpopulation of T helper cells is involved in the development and sustaining of germinal center (GC) interactions, an essential crosstalk that promotes the generation of long-lived high-affinity humoral immunity. Since the interaction between Tfh and B cells is mediated both by cell-associated and soluble factors, including CD154 (CD40L), ICOS, IL-21, IL-10, and IL-4 [52], the Env-specific CD4^+^ Tfh cell responses were studied analyzing those parameters by ICS assay in splenocytes obtained from immunized mice at 10 days after the last immunization. Thus, splenocytes were non-stimulated (RPMI) or stimulated ex vivo for 6 h with HIV-1 gp120_BX08_ protein plus Env peptide pool. Frequencies of total CD4^+^ T cells with Tfh phenotype (CXCR5^+^, PD1^+^) were significantly higher in animals immunized with DNA-gp120/MVA-LEO160-gp120 than in those immunized with DNA-gp120/MVA-B; in both cases, the frequencies were lower than in animals of control group DNA-ϕ/MVA-WT (Figure 6A), probably due to an immunosuppressive function of the HIV-1 Env protein. Afterwards, the HIV-1 Env-specific Tfh response was evaluated by quantifying the percentage of CD4^+^ Tfh cells that produced CD154 and/or IL-21 and/or IL-4. Since about 70% of the CD4^+^ Tfh cells obtained in the non-stimulated (RPMI) or stimulated (with the gp120_BX08_ protein plus the Env peptide pool) conditions were positive for IL-21, whereas in the CD4^+^ non-Tfh population only 2% of the cells were IL-21^+^, the Env-specific Tfh response was finally established by analyzing the percentage of CD4^+^ Tfh cells that produced CD154 and/or IL-4 after stimulation, in comparison with non-stimulated cells (Figure 6B). The results showed that the magnitude of the HIV-1 Env-specific Tfh response induced by animals immunized with DNA-gp120/MVA-LEO160-gp120 was significantly higher than in animals immunized with DNA-gp120/MVA-B (Figure 6B).

### 3.6. MVA-LEO160-gp120 Enhances the Levels of Antibodies against HIV-1 gp120

**Figure 7:**
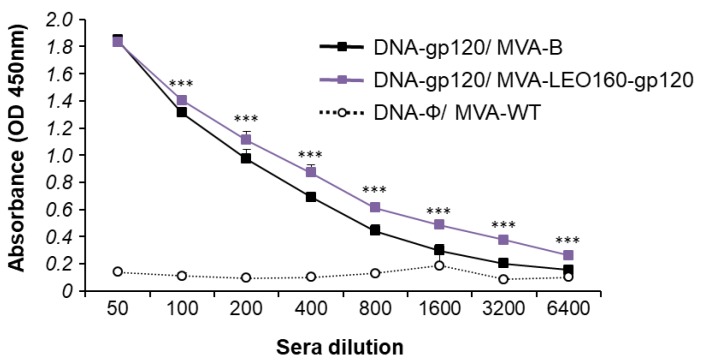
Humoral immune responses elicited by DNA-gp120/MVA-B and DNA-gp120/MVA-LEO160-gp120 against HIV-1 gp120 protein. Levels of gp120-specific total IgG binding antibodies were measured by ELISA in pooled sera from mice immunized with DNA-gp120/MVA-B, DNA-gp120/MVA-LEO160-gp120 or DNA-ϕ/MVA-WT (n = 5) 10 days after the last immunization. Mean absorbance values (optical density (OD) measured at 450 nm) and standard deviations of duplicate pooled serum dilutions are represented. *P* values indicate significant differences in antibody levels between the DNA-gp120/MVA-B and DNA-gp120/MVA-LEO160-gp120 immunization groups at each serum dilution (***, *p* < 0.001).

Since both the cellular and humoral arms of the immune system are thought to be necessary to control HIV-1 infection [53], the humoral responses elicited after immunization with DNA-gp120/MVA-B and DNA-gp120/MVA-LEO160-gp120 were also analyzed, quantifying by ELISA the total IgG levels of antibodies against HIV-1 gp120 protein (clade B, isolate BX08) in pooled sera obtained from mice 10 days post-boost (Figure 7). The results showed that DNA-gp120/MVA-LEO160-gp120 elicited higher levels of total IgG anti-gp120 antibodies than DNA-gp120/MVA-B. While the differences observed were small, they might have biological relevance.

## 4. Discussion

One of the approaches more recently applied to increase the immunogenicity of MVA recombinant vectors is the use of stronger VACV promoters to enhance the levels of expression of foreign antigen(s) inserted in the MVA genome [18,54]. Poxviral promoters contain different sequence motifs that can be classified into early, intermediate, and late, depending on the expression timing during poxvirus infection [55,56]. The modification of the promoter sequence is an excellent approach to control transgene expression; for example, some have both early and late elements, allowing their open reading frames or recombinant antigens to be expressed early in the virus infection and late after the viral genome replication. In the last few years, a number of poxviral promoters have been tested in recombinant MVA vectors, to increase recombinant antigen expression and, potentially, enhance antigen-specific immune responses [19,20,29,30,57,58]. Among them, one of the most promising is the novel synthetic VACV LEO promoter, which was previously designed in our laboratory using bioinformatic approaches and contains a late motif followed by an optimized immediate-early motif that allowed the transcriptional control of a heterologous antigen. The LEO promoter enhanced GFP expression and the magnitude of GFP-specific CD8^+^ T cells in immunized mice [30]. Further improvement of the LEO promoter was achieved by elongating from 38 to 160 nucleotides the spacer sequence between the promoter elements and the transgene transcriptional start site (termed LEO160 promoter), thus improving antigen-specific memory CD4^+^ and CD8^+^ T cell responses in immunized mice, as tested with GFP or the Leishmania antigen LACK [29]. Thus, considering the strength of the LEO160 promoter in inducing better early expression of the intracellular GFP and LACK antigens, and improving antigen-specific cellular immune responses in immunized mice, this promoter modification was introduced in the context of an MVA-based HIV/AIDS vaccine candidate. Therefore, insertion of the novel VACV optimized LEO160 promoter was introduced in MVA to try to enhance the expression and immunogenicity of the HIV-1 gp120 antigen from clade B. The recombinant virus MVA-LEO160-gp120 was generated with the aim to define the role of the LEO160 promoter strength in the early expression and secretion of the soluble HIV-1 gp120 antigen, and to test whether gp120-specific cellular and humoral immune responses could be enhanced.

When a new promoter or insertion site is introduced within the genome of any viral vector, the demonstration of the genetic stability of the transgenes should be tested, because several reports have suggested that the promoter used and/or the insertion site could affect the stability of the recombinant transgene [59,60,61]. Although genetic stability data were absent in previous LEO promoter reports [29,30], here the high genetic stability of MVA-LEO160-gp120 is demonstrated through long-term passages in cell culture showing high levels of expression of the HIV-1 gp120 antigen during all the passages.

The temporal expression of HIV-1 gp120 under the control of the LEO160 promoter was studied and the results showed that the mRNA transcription levels of HIV-1 gp120 and the total HIV-1 gp120 protein production in cells infected with MVA-LEO160-gp120 was significantly upregulated during most of the times studied, compared with cells infected with the HIV/AIDS vaccine candidate MVA-B, expressing the HIV-1_BX08_ gp120 under the control of the widely used sE/L promoter [62]. These results confirm the previous results obtained in our laboratory [29,30], and are in agreement with various recent studies reporting that new early promoters increase the expression of heterologous antigen under their transcriptional control [20,57,58], when compared to the early and late p7.5 promoter (p7.5), one of the first VACV promoters described [63], and to the widely used sE/L VACV promoter [62]. Moreover, the analysis of the HIV-1 gp120 secretion to the extracellular media showed a significant enhancement at early times post-infection in cells infected with MVA-LEO160-gp120, compared to MVA-B-infected cells; confirming that the LEO160 promoter can also enhance the cell release of a soluble antigen, such as HIV-1 gp120. Thus, the MVA-LEO160-gp120 vector enhanced the levels of intracellular and extracellular production of gp120 during infection, although there was no apparent difference between the vectors in the gp120 secreted levels at 6 and 24 h, probably due to the extensive cytopathic effect and induction of apoptosis triggered by the MVA vector. The higher levels of gp120 mRNA observed in cells infected with the MVA-LEO160-gp120 vector is likely due to the enhanced promoter strength, although increased mRNA stability and/or other cellular factors contributing to the stability can also be considered. Pulse-chase experiments of mRNA could help to define these differences.

Few comparative studies have reported on the choice of transgene promoter or insertion site and heterologous antigen secretion in vitro from poxviral vectors, but some reports have associated an increase in the secretion of an MVA transgene with an enhanced transgene-specific immune responses [64]. Thus, to determine whether the enhanced levels of HIV-1 gp120 expressed by MVA-LEO160-gp120 observed in cultured cells correlate with an increased magnitude of HIV-1-specific T cellular and humoral immune responses in vivo, a DNA-gp120 prime/MVA boost immunization protocol was performed in mice, as this heterologous regimen has been shown to increase the antigen-specific T cell and humoral immune responses over homologous immunization vectors [4,32]. Compared to DNA-gp120/MVA-B, DNA-gp120/MVA-LEO160-gp120 significantly enhanced the magnitude of the adaptive HIV-1 gp120-specific CD4^+^ and CD8^+^ T cell immune responses. Similar results were obtained using a homologous MVA/MVA immunization regimen but, as expected, the elicited HIV-1 gp120-specific CD4^+^ and CD8^+^ T cell responses magnitudes were lower. Apart from the results obtained from previous reports of our lab with the novel LEO promoter, many other reports confirmed a positive correlation between enhanced early expression triggered by MVA vectors and increased T cellular immunogenicity [19,20,57,59]. In particular, a previous report of MVA recombinants expressing either enhanced GFP or chicken ovalbumin, each under the control of a hybrid early–late promoter (pHyb) compared with the widely used p7.5 and sE/L promoters, have demonstrated that a stronger immediate-early neoantigen expression by a poxviral vector results in superior induction of neoantigen-specific CD8^+^ T cell responses [20], and were able to stimulate potent recall responses after repeated boosters providing an advantage in the context of homologous vaccination regimes and immunotherapy [57]. Furthermore, in the field of MVA-based HIV/AIDS vaccine candidates, a previous report has already correlated a 4- to 7-fold enhanced expression of HIV-1 Env antigen driven by the strong mH5 promoter with a significant increase in Env-specific CD4^+^ (1 to 2-fold) and CD8^+^ T (3- to 5-fold) cell responses [19]. These results are in agreement with the results obtained with the MVA-LEO160-gp120, in which up to 9-fold enhanced expression of HIV-1 gp120 antigen correlated with an increase in Env-specific CD4^+^ (1.5-fold) and CD8^+^ T (3-fold) cell immune responses, and highlights that the novel LEO160 promoter appears superior to other widely used viral and synthetic promoters, such as p7.5. Moreover, we have also previously described that the LEO promoter was superior to sE/L promoter in terms of increased early antigen expression and of antigen-specific CD8^+^ T cell immune responses [29,30].

The increase in HIV-1 gp120-specific CD4^+^ and CD8^+^ T cell responses obtained with the DNA-gp120/MVA-LEO160-gp120 immunization could be of relevant importance, because several studies indicate that the HIV-1-specific cellular response goes some way towards controlling HIV-1 infection, although it fails ultimately to deal with virus infection [65]. Vaccines that can stimulate both CD4^+^ and CD8^+^ T cell responses to HIV-1 may be able to control the virus early in infection before the virus causes major immune damage, as was demonstrated with the partial efficacy obtained in the RV144 trial [9].

Additionally, when evaluating the HIV-1-specific cellular immune responses, it is also important to consider the memory phenotype of the T cells elicited, because a fast acquisition of TEM and TE phenotypes in the adaptive phase could be important in the development of the T cell memory responses and in the mounting of a more effective immunity during a primary pathogen encounter, as the presence of TEM cells has been correlated with protection in the macaque-SIV model [66,67]. In this work, both immunization groups elicited mainly HIV-1 gp120-specific T cells of a TEM phenotype, followed by a TE phenotype; again, DNA-gp120/MVA-LEO160-gp120 significantly enhanced the magnitude of these T cell populations, which is a positive cell marker for HIV-1 protective responses.

Furthermore, a CD4^+^ T cell population has been identified, named Tfh cells, which is responsible for providing help to B cells [68]. Since then, a deep research of this T cell subpopulation has been done in the context of HIV-1 infection and vaccine development [51]. Circulating HIV-1-specific IL-21^+^ Tfh cells were found at higher frequencies in sera from participants in the partially protective ALVAC+AIDSVAX (RV144) HIV/AIDS clinical trial compared to the non-protective DNA+Ad5 clinical trial, thus correlating protective antibody responses with elevated percentages of this CD4^+^ T cell subtype [69]. Moreover, in HIV-1-infected patients, a correlation between the frequencies of circulating Tfh cells and the induction of bNAbs has been reported [70], and in HIV-1 controllers, higher percentages of circulating Tfh cells have been associated with the induction of HIV-1-specific antibodies in functional assays favoring preserved memory B cell responses [71]. Given the central role for the Tfh cell response in inducing protective responses against HIV-1, the percentages of total and HIV-1 gp120-specific Tfh cells elicited by the recombinant MVA-LEO160-gp120 in comparison with the MVA-B vaccine candidate was studied. The results obtained in immunized mice showed that the overall magnitude of HIV-1-gp120-specific Tfh cell response was significantly higher in splenocytes from animals receiving DNA-gp120/MVA-LEO160-gp120 immunization compared with the group immunized with DNA-gp120/MVA-B, disclosing the ability of the LEO160 promoter to increase also the HIV-1-gp120-specific response of this important cell subtype. Since the differences observed were small, future experiments will be performed to further characterize in detail the impact of the novel MVA-LEO160-gp120 vector on Tfh cell responses. These findings are in agreement with recent results from our laboratory that suggest that MVA-based vectors might represent an advantageous platform to potentially activate HIV-1-specific Tfh cell responses [44,45].

Although the positive correlation between the heterologous antigen expression in the MVA system and the improvement of the T cell (particularly CD8^+^) responses has been well documented [19,20,29,57,59], none of these reports have found difference in the levels of neo-antigen-specific antibodies independently of the promoter used [57]. This result was attributed to the fact that during VACV infection, the late and intermediate genes have shown to be the preferred targets for antibody responses [21,22], but the factors that regulate and determine the antibody responses from MVA expressed genes are still not well defined. Here, when HIV-1-specific humoral immune responses elicited in serum samples from immunized animals were analyzed by ELISA, the data revealed that DNA-gp120/MVA-LEO160-gp120 immunization protocol enhanced the levels of total IgG binding antibodies against HIV-1 gp120 protein compared to DNA-gp120/MVA-B. While the differences observed between both groups were small, they might be biologically relevant. The more efficient production of anti-Env-specific antibodies seen may be due to the higher expression of HIV-1 Env in infected cells, as this slightly higher total IgG HIV-1 Env binding antibody levels observed in mice immunized with DNA-gp120/MVA-LEO160-gp120 is consistent with the higher levels of gp120 observed in MVA-LEO160-gp120-infected cells. Even though it is suggested that in MVA immunizations antibodies are mainly induced against late poxviral antigens [72], here we observed an enhancement in the antigen-specific antibody responses by an early strong transgene expression. Although promoter optimization within the VACV replicative strain LC16m8 expressing HIV-1 Env was found to increase production of anti-HIV-1 Env-specific antibodies when the stronger SFJ1-10 promoter was used, compared with the widely used p7.5 promoter [58], this is the first time that this phenomenon is described for a recombinant MVA vector. Higher levels of total IgG in serum could be an important parameter associated with the protective effect induced by HIV/AIDS vaccine candidates, because studies on the RV144 vaccine regimen revealed that the protection against HIV-1 infection was directly correlated with the level of IgG antibodies specific for the HIV-1 gp120 V1V2 region [9,73]. Future studies should aim to analyze in detail the humoral immune responses elicited by this novel LEO160 promoter, such as the induction of different isotypes, peptide mapping, neutralizing antibodies, and antibody-dependent cellular cytotoxicity.

## 5. Conclusions

In summary, the results obtained demonstrate how a designed VACV promoter modification can be used to enhance the levels of HIV-1 gp120 soluble protein in cultured cells infected with an MVA vector. In mice, the magnitude of the HIV-1 gp120-specific CD4^+^ and CD8^+^ T cell immune responses and the levels of anti-gp120 antibodies were also increased, demonstrating the enhanced immune properties of this promoter. Thus, based on its capacity to increase heterologous antigen expression in vitro and antigen-specific CD4^+^ and CD8^+^ T cell responses in vivo, the novel synthetic VACV LEO160 promoter is a promising prototype to be used in the generation of poxvirus-based vaccine vectors.
